# Digital medicine: the next big leap advancing cardiovascular science

**DOI:** 10.1186/s12872-022-02971-5

**Published:** 2023-01-17

**Authors:** Alexander Kharlamov, Morten Lamberts

**Affiliations:** 1 Advanced Cardiovascular Imaging Lab, De Haar Research Foundation (DHRF), Tallinn, Estonia; 2 Innovation Lab, De Haar Research Task Force, Rotterdam, The Netherlands; 3 Department of Clinical Medicine, University of Copenhagen, Copenhagen, Denmark; 4 Department of Cardiology, Herlev-Gentofte University Hospital, Herlev, Denmark; 5 DHRF, Keurenplein 41, G9950, 1069 CD Amsterdam, The Netherlands

## Abstract

Solid clinical and academic leadership remains necessary to ensure that healthcare based on digital technologies is relevant, meaningful, and stands on the best possible evidence. This compendium accompanying the “Digital Technologies in Cardiovascular Disorders” article collection in *BMC Cardiovascular Disorders* summarizes recent knowledge about robust and advanced digital tools for preventing, monitoring, diagnosing, and treating cardiovascular diseases.

The fruits of digital medicine are almost ripe and will unequivocally become prominent in modern clinical medicine. In the *Digital Technologies in Cardiovascular Disorders* article collection in *BMC Cardiovascular Disorder*s, we present articles published in the journal between 2021–2022 with a focus on digital medicine, elaborating on recent progress in the field, as well as the relevance of the accomplishments to the routine clinical practice, and the challenges modern-day technology faces today.

Many international institutions consider digital medicine as a tool for transforming healthcare. The EU4Health program 2021–2027 of the European Commission [1] pursues four general objectives representing the ambitions of the program and ten specific objectives, amongst them strengthening healthcare systems through reinforcing health data, digital tools, and services, as well as the digital transformation of healthcare. Due to rapidly growing interest in the field, the DigitalHealthEurope initiative was created, which is a Co-ordination and Support Action that supports advancing the initiatives outlined in the European Commission’s Communication on the Digital Transformation of Health and Care [1].

Meanwhile, it should be noted that despite the particular success, this area of scientific knowledge continues to be a twilight zone due to the relative imperfection of technology and, perhaps, caution from clinicians embracing new technology. Indeed, digital technology remains extremely operator-dependent, adversely affecting the studies' accuracy and implementability. The volume and quality of clinical trials also remain insufficient for certain technologies to be included in official clinical guidelines. Some new technologies require alternative methodological and statistical approaches. Whether we can trust the results from such trials to translate findings into daily caregiving in clinical, organizational, or policy attitudes remain unanswered. Therefore, more extensive clinical trials need to be set up. At the same time, digital medicine has a vast potential that should not be underestimated [2, 3]. In particular, the automation of physician diagnostics (including advanced cardiovascular imaging and challenges of genetics) or therapy-related information is critical today for inpatient daily clinical practice. Moreover, the new types of smart devices and sensors can significantly increase the quality of outpatient home diagnostics. Regardless of the diagnostic setting, these developments provide the possibility of an optimized individual approach to treatment.

Patient-generated data, including wearables, home monitoring of blood pressure, pulse, electrocardiogram, sleep parameters, pulse oximetry, and many others, may modify our health awareness [2, 3]. Such individualized interventions might be used for remote and home health monitoring, revealing daily physiological patterns and life-threatening or urgent conditions. They can provide caregivers with high-accuracy valuable clinical information [4]. In the mixed-methods observational study of A’Court et al. [4], the research group sought to quantify instances of patient-initiated home monitoring (collected with, for example, blood pressure monitors, pulse oximeters or internet-connected devices) as reported in letters by clinicians, characterize the contribution of patient-generated data (e.g., blood pressure monitoring, wearables, KardiaMobile, pulse oximeter, home 12-lead ECG, stopwatch, bike monitor, iPhone pulse monitor) to clinical decision-making, and explore clinical experiences and views of self-monitoring practices. The self-monitoring devices contributed to clinical decision-making or management in 69% of cases. Data was successfully used in 25% of cases to make cardiac diagnoses (mainly for narrow complex tachyarrhythmias and hypertension). This evidence suggests that digital technology benefits clinical decision-making, ultimately demonstrating its potential to enrich routine clinical practice and therefore reduce morbidity, improve outcomes and diminish mortality in cardiovascular patients.

Undoubtedly, the last decade's progress cannot be ignored and is already beneficial nowadays against all odds, especially in machine learning (ML)-based advanced cardiovascular imaging for noninvasive and invasive cardiology needs. The developments of coronary computed tomography angiography (CCTA) and cardiac magnetic resonance (CMR) already became a gold standard approach for diagnosing coronary artery disease, especially in the case of chronic coronary syndrome or tailoring the clinical strategy in acute care, vulnerable and stable patients [5]. Noninvasive imaging can provide a caregiver with information about coronary anatomy, localization, degree of stenosis, type of lesion, the volume of the associated ischemia, and the impaired function of the myocardium. This critical data is necessary for optimizing any subsequent pharmaco-invasive strategy [6]. Novel imaging techniques in the invasive stage bear a solution for intervention with higher accuracy and safety. In the retrospective analysis (269 vessels in 141 patients) of Lossnitzer et al. [7], the diagnostic performance of machine-learning computed tomography-based fractional flow reserve (CT-FFRML) compared to stress perfusion cardiovascular magnetic resonance (CMR) was examined, evaluating whether there is an additional value of CT-FFRML over CCTA. The univariate logistic regression analysis demonstrated a significant association between CT-FFRML (mainly for a stenosis ≥ 70%) and stress perfusion CMR with Chi2 = 47 (*p* < 0.0001). CCTA with ≥ 50% stenosis could not achieve the level of significance upon Chi2 testing. Ischemia remains a critical criterion for deciding on optimal invasive strategy in patients with chronic coronary syndrome. The revelation of both significant artery stenosis with CCTA and confirmed ischemia in the specific coronary pool with CMR has promising potential for an individualized invasive approach. These results agree with the SCOT-HEART (NCT01149590), PACIFIC (NCT01521468), ICONIC (the CONFIRM registry), PARADIGM (NCT02803411), CREDENCE (NCT02173275), and PROMISE (NCT01174550) trials underlying benefits of CCTA and exposing the valuable role of CMR in patients with chronic CAD [7]. In the future, the development of software can allow the merging of advanced data from CCTA, CMR, and positron emission tomography analyses into a single coronary imaging report, providing comprehensive information about coronary anatomy, type of lesions, blood perfusion and function of the myocardium with the assessment of the associated cardiovascular risk. Increased knowledge on coronary status through multimodality imaging may better identify stable patients not needing invasive interventions, but, importantly, the clinical effects need to be tested in a randomized setting. Indeed, the landmark ISCHEMIA trial (NCT01471522) [7] failed to show invasive superiority despite moderate and severe ischemia.

Modern medicine is on the verge of a new revolution, already integrating digital products into clinical practice [3–7]. This impending furtherance can significantly speed up and improve the quality of diagnostics in the future, thus ensuring timely medical care to patients and likely in a more cost-effective manner. Moreover, digital medicine will be able to provide valid and swift interpretation of diagnostic data, leading to the adoption of optimal treatment strategies. Remote monitoring tools and an optimized prevention scheme, including precision medicine data, will contribute to correctly identifying risk factors and creating practical approaches to improve preventive measures [8–10]. The massive whole-genome sequencing data arrays handled with artificial intelligence (AI) or ML software drive clinical medicine to a paradigm shift toward precision cardiology [3, 9]. ML, big data statistics, and advanced analytics accelerate development and create novel methodologies and solutions for daily clinical practice.

Paying attention to the development of AI and associated ML, that is, the processing and systematizing medical data, is vital [3]. Notwithstanding, AI technology for broader medical purposes is still in its infancy. The modern accomplishments in neurophysiology, biophysics, and mathematics do not allow brain emulation even of one person, which means that a genuine breakthrough in technological development should be expected only in a few decades, taking into account the current pace of digital evolution [11]. Such a breakthrough is impossible without the synergetic interaction of specialists in various fields within biomedical science and, first of all, in close collaboration with physicists, technologists, and engineers. Nonetheless, at this very moment, hyperconnected, predictive, and proactive solutions for AI are still missing.

Thus, there is no doubt that maturing digital medicine is a platform for advancing biomedical research, improving healthcare systems, and lower public health-related costs in the sense of greater productivity of science, operational efficiency of physicians, fulfilling the demands of patients for faster and more personalized care, reducing, therefore, morbidity rates, and creating the prerequisites for rocketing the duration and quality of life and, in general, a substantial reduction in mortality rates.

## Abbreviations

CCTA: Coronary computed tomography angiography
CT-FFRML: Machine-learning computed tomography-based fractional flow reserve
CMR: Cardiovascular magnetic resonance
CMR-FT: CMR feature-tracking
BP: Blood pressure
HBPM: Home blood pressure monitoring
ECG: Electrocardiogram
AI: Artificial intelligence
CAD: Coronary artery disease
